# Genetic (idiopathic) epilepsy with photosensitive seizures includes features of both focal and generalized seizures

**DOI:** 10.1038/s41598-018-24644-0

**Published:** 2018-04-19

**Authors:** Jiao Xue, Pan Gong, Haipo Yang, Xiaoyan Liu, Yuwu Jiang, Yuehua Zhang, Zhixian Yang

**Affiliations:** Department of Pediatrics, Peking University First Hospital, Beijing, China

## Abstract

Clinically, some patients having genetic (idiopathic) epilepsy with photosensitive seizures were difficult to be diagnosed. We aimed to discuss whether the genetic (idiopathic) epilepsy with photosensitive seizures is a focal entity, a generalized entity or a continuum. Twenty-two patients with idiopathic epilepsies and photoconvulsive response (PCR) were retrospectively recruited. In the medical records, the seizure types included “generalized tonic-clonic seizures (GTCS)” in 15, “partial secondarily GTCS (PGTCS)” in 3, partial seizures (PS) in 3, myoclonic seizures in 2, eyelid myoclonus in one, and only febrile seizures in one. Seizure types of PCR included GTCS (1/22), PGTCS (6/22), PS (9/22), electrical seizures (ES) (3/22) and GTCS/PGTCS (3/22). Combined the medical history with PCR results, they were diagnosed as: idiopathic (photosensitive) occipital lobe epilepsy (I(P)OE) in 12, genetic (idiopathic) generalized epilepsy (GGE) in one, GGE/I(P)OE in 5, pure photosensitive seizure in one, and epilepsy with undetermined generalized or focal seizure in 3. So, the dichotomy between generalized and focal seizures might have been out of date regarding to pathophysiological advances in epileptology. To some extent, it would be better to recognize the idiopathic epilepsy with photosensitive seizures as a continuum between focal and generalized seizures.

## Introduction

Photosensitivity is defined as an abnormal clinical and/or electroencephalographic (EEG) response evoked either by intermittent photic stimulation (IPS) or by visual stimuli in daily life^1,2^. Examples of environmental photic stimulation include electronic flicker (usually television), strobe light, and flickering sunlight^3^, which is easy to neglect unless specially inquired. As the population has been exposed to dramatically increased trigger stimuli in the modern age, photosensitivity is of increasing concern.

Seizures triggered by visual stimuli occur in up to 10% of epilepsies in childhood^4^. The term photosensitive epilepsy is not an epilepsy syndrome per se, it refers to a heterogeneous group of epileptic conditions characterized by photic- or pattern-induced seizures (video games, flicker, TV, color modulation, IPS, et cetera)^5,6^. In pure photosensitive epilepsy, seizures exclusively occur in response to photic stimulation as opposed to epilepsy with photosensitive seizures, where seizures may be spontaneous and elicited by photic stimulation^5,6^. The former includes idiopathic photosensitive occipital lobe epilepsy (IPOE) and photosensitive myoclonic epilepsy of infancy (MEI); the latter includes Dravet syndrome, Jeavons syndrome (JS), juvenile myoclonic epilepsy (JME) and so on^6,7^. In epilepsy with photosensitive seizures, epileptiform EEG responses induced by IPS with or without associated clinical symptoms, so-called photoconvulsive response (PCR) or photoparoxysmal response (PPR) respectively, could be observed^8^. As reported previously, seizure types associated with epilepsy with photosensitivity predominantly included generalized tonic-clonic seizures (GTCS), absences and myoclonic seizures^9^; while partial seizures (PS) were rarely encountered^10^. However, in the clinical practice, we noticed that most of the PCRs had electrographic seizures with or without clinical evidence to support a partial onset, particularly an occipital onset, no matter whether generalized or focal epileptic syndromes the patients were diagnosed according to medical histories. This phenomenon brought some divergence to the clinical diagnosis. In order to illuminate the role of photic-induced seizures on the diagnosis of photosensitive epileptic syndromes and discuss the possible mechanisms of photosensitivity, we retrospectively screened a cohort of patients from the thousands of video-EEG (VEEG) recordings in the past few years. All these patients were considered as having idiopathic epilepsies and had PCRs, the latter including primary or secondary GTCS, PS and also electrical seizures (ES) similarly to the ictal pattern of PS.

## Patients and Methods

### Ethics Statement

This study was approved by the Biomedical Research Ethical Committee of Peking University First Hospital, and written informed consents were obtained from the legal guardians (parents) of the children. All experiments were performed in accordance with relevant guidelines and regulations. All data were analyzed anonymously.

### Patients

**Figure 1:**
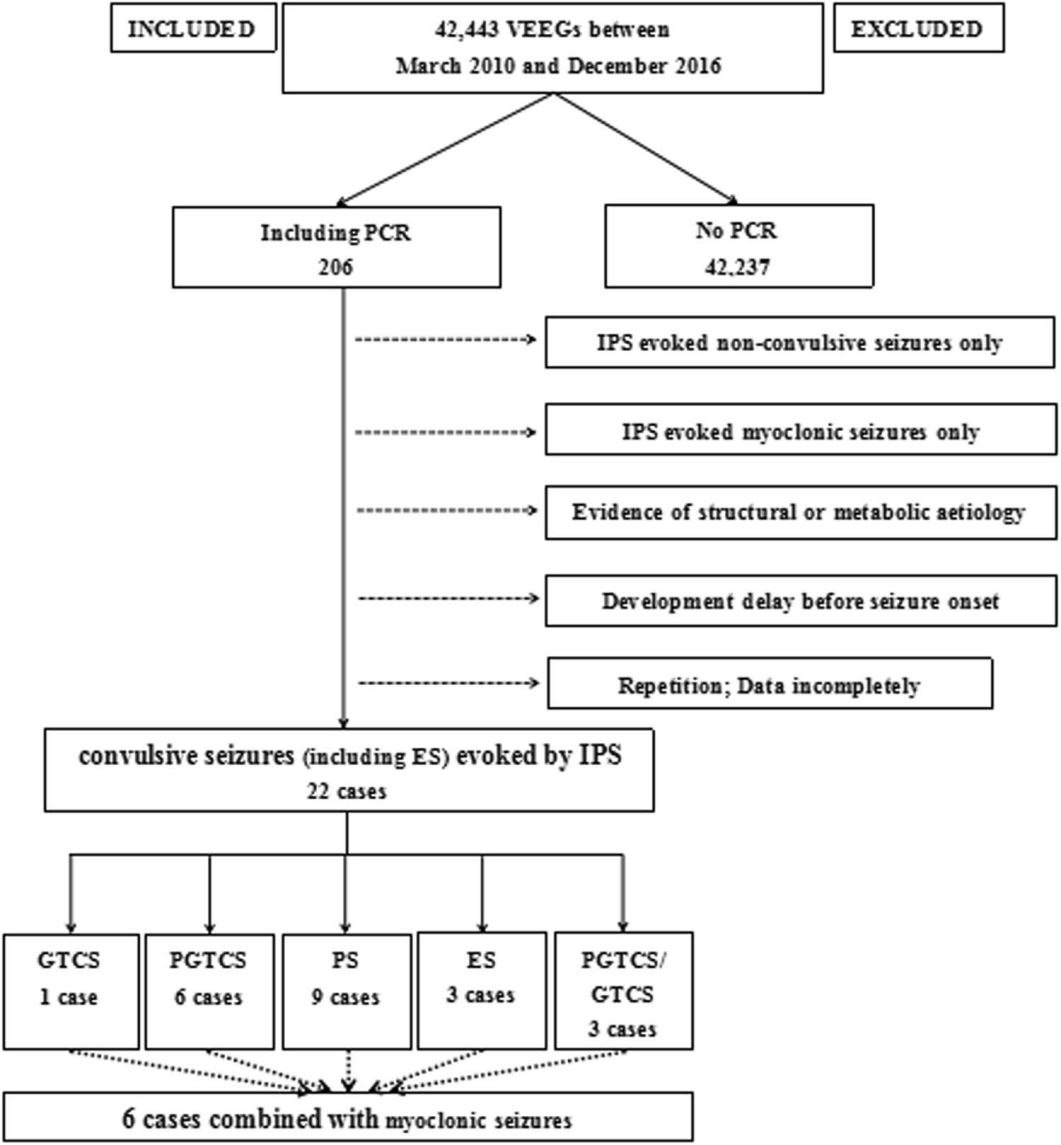
The screening diagram of 22 patients included in the study. VEEG: video electroencephalography; PCR: photoconvulsive response; IPS: intermittent photic stimulation; GTCS: generalized tonic-clonic seizures; PGTCS: partial secondarily generalized tonic-clonic seizures; PS: partial seizures; ES: electrical seizures.

Twenty-two children were retrospectively recruited from approximately 42,443 VEEG recordings monitored in our hospital between March 2010 and December 2016 (Fig. 1). Inclusion criteria: 1) IPS evoked seizures including GTCS, partial secondarily GTCS (PGTCS), PS, or ES were identified by VEEG. ES was defined as EEG seizure pattern similarly to the ictal pattern of PS but not accompanied by clinical manifestations and was classified as subclinical seizure activity. 2) An idiopathic etiology was considered according to the normal findings on neurological examination, metabolic screening, magnetic resonance imaging (MRI), and development before seizure onset.

### VEEG monitoring

VEEG monitoring was performed using a Nihon Kohden digital video-EEG-1100 K instrument for about 4 hours, covering both awake and sleep states. EEG electrodes were positioned over the scalp according to the international 10–20 system, with the corresponding polyelectromyography to record the activities of muscles including deltoid, quadriceps femoris and so on. EEG and PEMG activities were recorded with bandpasses of 0.3–70 Hz and 5.3–120 Hz. In wake state, open-close eyes test, hyperventilation and IPS were performed in all patients. According to an international standard in combination with our practical application^11,12^, IPS was performed in a dimly lit environment, using a round lamp with 10 cm in diameter. The distance between the patient and the lamp was about 30 cm. We used a continuous sweep of rising frequencies including 1, 2, 4, 6, 8, 10, 12, 14, 16, 18, 20 Hz and falling frequencies including 60, 50, 40, 30, 25 Hz. Ten-second trains of flashes for each frequency were delivered, at intervals of 10 seconds. Stimulation was delivered with the patient’s eyes opened, eyes closed and eyes closure in turn. Under conditions of eyes opened and eyes closed, the patients keep their eyes opened or eyes closed during the whole constant photic frequencies. Under the condition of eyes closure, the patients closed their eyes immediately after each photic frequency began and opened their eyes after each photic frequency was just over. If seizure was evoked, IPS was terminated immediately. Diagnosis of photosensitivity was made if localized or generalized spike or spike-and-wave activities were evoked by IPS. The EEG traces were evaluated by two qualified neurophysiologists.

### Data collection

Clinical records and VEEG recordings were reviewed to obtain information including sex, history of febrile seizures, age at seizure onset, seizure types, electroclinical features, treatment, as well as family history of epilepsy or febrile seizures. Psychomotor development was evaluated clinically in all patients.

## Results

### General features

Twenty-two patients (8 males and 14 females) were identified. The mean and median ages at seizure onset were 6.3 and 5.5 years (range: 1–13 years). Eight patients (36.4%) had a history of febrile seizures. Five patients (22.7%) had a family history of epilepsy, and two patients (9.1%) had a family history of febrile seizures. The detailed information was summarized in Table 1.

**Table 1:** General characteristics of the patients in our study.

No	Sex	Age of onset	Age at EEG monitoring	AEDs used before EEG monitoring	AEDs at EEG monitoring	History of FS	Family history of epilepsy or FS
1	F	4 y	5y5m	—	—	—	—
2	F	12 y	12 y	—	—	—	—
3	M	2y6m	17 y	PB, VPA	VPA	+	—
4	M	6 y	8 y	VPA	—	—	EP
5	F	2y6m	6 y	LEV, VPA, NZP, LTG	VPA, LTG	—	—
6	M	13 y	14 y	—	—	—	EP
7	M	12 y	14 y	VPA	VPA	—	—
8	M	6 y	11 y	VPA, PB, CBZ	VPA, PB, CBZ	—	—
9	F	10 y	11 y	—	—	—	FS
10	F	12 y	14 y	LEV	LEV	—	—
11	F	6 y	16 y	VPA, LTG	LTG	—	EP
12	F	1 y	2y6m	LEV, CZP, VPA, LTG, TPM	LEV, CZP	+	EP
13	F	5 y	10 y	—	—	+	—
14	F	6 y	8 y	—	—	—	—
15	M	11 y	11 y	—	—	—	EP
16	F	5 y	10 y	VPA	—	—	—
17	M	2 y	13 y	VPA, LTG	—	—	FS
18	F	4y4m	5 y	—	—	+	—
19	F	8 y	8 y	—	—	+	—
20	F	5 y	9 y	OXC	OXC	+	—
21	M	2y6m	4y2m	LEV, VPA	LEV	+	—
22	F	3y6m	6 y	CBZ, VPA, LTG	OXC, VPA	+	—

AEDs: antiepileptic drugs; FS: febrile seizures; EP: epilepsy; PB: phenobarbital; VPA: valproic acid; LEV: levetiracetam; NZP: nitrazepam; LTG: lamotrigine; CBZ: carbamazepine; CZP: clonazepam; TPM: topiramate; OXC: oxcarbazepine.

### Clinical manifestations

We carefully reviewed the seizure symptoms recorded in the medical records and roughly classified the seizure types as follows (Table 2): “GTCS” (quotation marks for lacking identification by VEEG) in 15 patients, manifesting as limbs convulsions without preceding focal symptoms and with loss of consciousness; “PGTCS” in 3 patients (patient 2, 13, 17), manifesting as consciously eyes or head deviation (left or right) followed by limbs convulsions and loss of consciousness; PS in 3 patients (patient 13, 18, 20), alone in two and combined with “PGTCS” in one, manifesting as consciously eyes and/or head deviation (left or right), nausea or some sensory symptoms such as mind blank or indescribable discomfort, without secondary limbs convulsions; myoclonic seizures in 2 patients, alone in patient 14 and combined with “GTCS” in patient 12, manifesting as a sudden shake of the whole body or only single or multiple limb(s), or a rapid nod; eyelid myoclonus induced by light stimuli combined with “GTCS” in patient 5. Patient 9 had fear of strong light in daily life. The frequency of seizures varied among patients, from 3–4 times a year to once every couple years for “GTCS”, “PGTCS” and PS, and several times per day for myoclonic seizures. In addition, only febrile seizures were observed in patient 22.

**Table 2:** IPS features of the patients in our study.

No.	Seizure type by history	Seizure frequency	Interictal focal SW	Interictal GSW	PPR					Eyes state of PCR (Hz)	Seizure type of PCR	Diagnoses	
					posterior SW	GSW	EO (Hz)	EC (Hz)	ECL (Hz)			history	history +EEG
1	“GTCS”	several times/y	Bilateral occipital SW	+	+	+	10–60	/	/	EO (30)	MS-PS	GGE	GGE/I(P)OE
2	“PGTCS”	2 events	—	Posterior prominent	—	+	10–60	—	/	EC (16)	MS-GTCS	IOE	GGE
3	“GTCS”	0–2/y	—	+	+	+	10–30	10, 6–25	10–18	ECL (18)	MS-ES	GGE	GGE/I(P)OE
4	“GTCS”	1/y	Rolandic SW	+	+	+	8–16	/	/	EO (16)	MS-PS	GGE	GGE/I(P)OE
5	“GTCS”, EM	1/several months, dozens of times/d	Left occipital SW	+	—	+	10–12	/	/	EO (12)	MS-PGTCS	GGE (JS)	GGE/I(P)OE
6	“GTCS”	2 events	—	+	—	+	6–30	/	/	EO (30)	MS-PGTCS/GTCS	GGE	undetermined
7	“GTCS”	3 events	Bilateral occipital SW	+	+	+	20	/	/	EO (30)	PGTCS/GTCS	GGE	undetermined
8	“GTCS”	1/1y-2y	—	+	—	+	8–12	/	/	EO (14)	PGTCS	GGE	I(P)OE
9	“GTCS”	3 events	Right posterior SW	—	—	—	—	/	/	EO (10)	PGTCS	GGE	I(P)OE
10	“GTCS”	1/0.5y-1.5 y	—	+	—	+	10–20	/	/	EO (25)	PGTCS	GGE	I(P)OE
11	“GTCS”	3–4/y	—	—	+	+	12–20	/	/	EO (30)	PGTCS	GGE	I(P)OE
12	“GTCS”, MS	1 event, several times/d	Bilateral occipital SW	—	—	—	—	/	/	EO (6)	PGTCS/GTCS	GGE (MEI)	undetermined
13	“PGTCS”, PS	1–2/y	Rolandic and anterior SW	+	+	+	14–18	/	/	EO (20)	PGTCS	IOE	I(P)OE
14	MS	several times/d	Rolandic and posterior SW	+	+	+	8, 12–30	6–60	6–60	ECL (30)	PS	GGE	GGE/I(P)OE
15	“GTCS”	2 events	Right posterior SW	+	—	+	6–10, 60	2–8, 20, 50	6, 14, 18	ECL (18)	PS	GGE	I(P)OE
16	“GTCS”	3–4/y	—	+	—	+	12–50	18–30	—	ECL (25)	PS	GGE	I(P)OE
17	“PGTCS”	1–2/y	—	+	—	+	6–20	10–25	8–30	EC (8)	ES	IOE	I(P)OE
18	FS, PS	3 events	Posterior SW, left prominent	+	—	—	—	—	—	ECL(25)	PS	IOE	I(P)OE
19	“GTCS”	1 event	Posterior SW	+	—	+	8–12	10–12	/	EC (12)	PS	GGE	I(P)OE
20	PS	1–2/y	Rolandic and occipital SW	+	+	+	2–30	8–20	4–20	ECL (20)	PS	IOE	I(P)OE
21	FS, “GTCS”	1–3/y	Bilateral occipital SW	—	+	+	6–12, 20–30	6, 12–20, 60	6–14	EC (6)	ES	GGE	I(P)OE
22	FS	1/y	—	+	+	+	14–20	—	/	EC(16)	PS	FS	Pure photosensitive seizure

IPS: intermittent photic stimulation; AEDs: antiepileptic drugs; SW: spike and waves; GSW: generalized spike and waves; EO: eye opened; EC: eye closed; ECL: eye closure; PPR: photoparoxysmal response; PCR: photoparoxysmal convulsion response; PS: partial seizures; GTCS: generalized tonic-clonic seizures; PGTCS: partial secondarily generalized tonic-clonic seizures; ES: electrographic seizures; EM: eyelid myoclonus; GGE: genetic (idiopathic) generalized epilepsy; IOE: idiopathic occipital epilepsy; JS: jeavons syndrome; MEI: myoclonic epilepsy of infancy; I(P)OE: idiopathic (photosensitive) occipital lobe epilepsy.

### VEEG data

The VEEG examinations were performed at the mean age of 9.8 years (range: 2.5–17 years) (Table 1). A normal background activity was observed in all patients. The interictal EEG showed generalized spike-and-wave alone in 8 patients, focal or multifocal spike-and-wave alone in 3 patients, both generalized and focal spike-and-wave in 10 patients. No interictal discharges were observed in one patient. In the 18 patients with interictal generalized discharges, the amplitude was gradually descending from anterior to posterior area in 17 patients, but higher amplitude in posterior area in one. In the 13 patients with interictal focal or multifocal discharges, the locations included unilateral or bilateral occipital region alone in 5, unilateral or bilateral posterior area alone in 4, Rolandic areas alone in one, overlapping between Rolandic areas and other locations in 3, including Rolandic and occipital areas in one, Rolandic and posterior areas in one, Rolandic and anterior areas in one.

**Figure 2:**
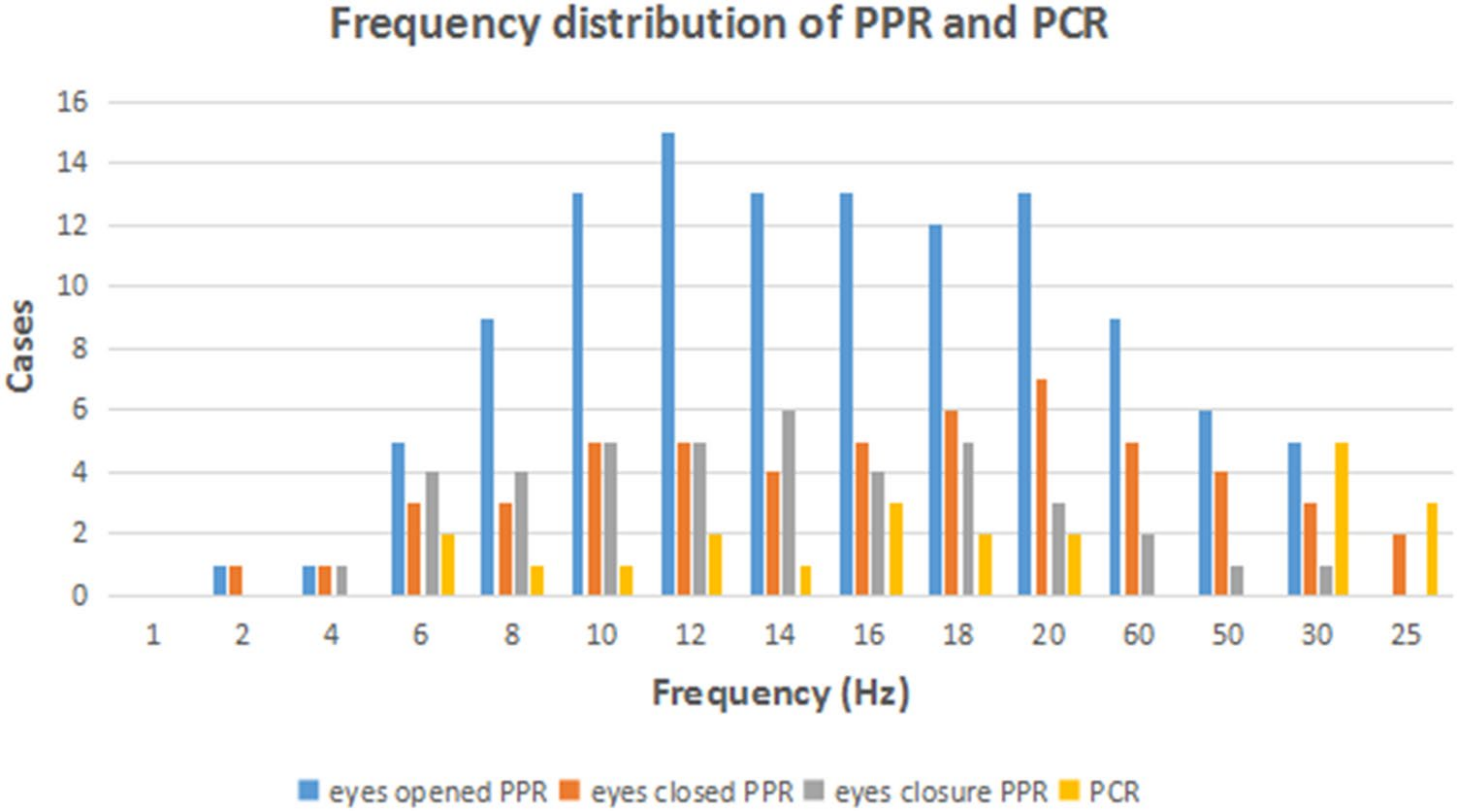
The frequency distribution of PPR and PCR. PPR: photoparoxysmal response; PCR: photoconvulsive response.

PPR was evoked by eyes opened IPS in 19 patients (22 patients receiving eyes opened IPS), by eyes closed IPS in 8 patients (11 patients receiving eyes closed IPS), and by eyes closure IPS in 7 patients (9 patients receiving eyes closure IPS) respectively. The electrical presentations of PPR showed generalized spike-and-waves alone in 9 patients, both generalized and posterior foal spike-and-waves in 10 patients. PCR was observed in all patients, which was evoked by eyes opened IPS in 11 patients, by eyes closed IPS in 5 patients, by eyes closure IPS in 6 patients. The frequency distribution of PPR and PCR was shown in Fig. 2. The susceptive frequency was mainly concentrated in the range of 10–20 Hz. Seizure types of PCR included GTCS (1/22), PGTCS (6/22), PS (9/22), ES (3/22) and GTCS/PGTCS (3/22, undetermined GTCS or PGTCS), which were combined with myoclonic seizures in 6 patients. Only 4 patients (patient 13, 17, 18, 20) had consistent partial seizures (PS/PGTCS/ES) in PCRs with the spontaneous PS or “PGTCS” described in the medical histories. However, 14 patients had inconsistent seizure types, including spontaneous “GTCS”, eyelid myoclonus or myoclonic seizures and IPS evoked PS/PGTCS/ES in 13 patients; and spontaneous “PGTCS” and IPS evoked GTCS combined with myoclonic seizures in one (patient 2). For patient 2 and other 5 patients (patient 5, 8–11) with spontaneous “GTCS” and IPS evoked PGTCS, their parents did not think there was any difference in the clinical presentations between the seizures in daily life and PCRs. Of the 18 patients with focal seizures in PCRs (PGTCS, PS or ES), 11 patients had interictal focal discharges, 9 patients had focal PPRs, and 4 patients had interictal generalized discharges and PPRs. For the patient 2 with GTCS of PCR, both the interictal discharges and PPRs were generalized. For the 3 patients with GTCS/PGTCS in PCRs, one (patient 6) had generalized interictal discharges and PPRs, one (patient 7) had both focal and generalized interictal discharges and PPRs, and one (patient 12) had focal interictal discharges alone.

**Figure 3:**
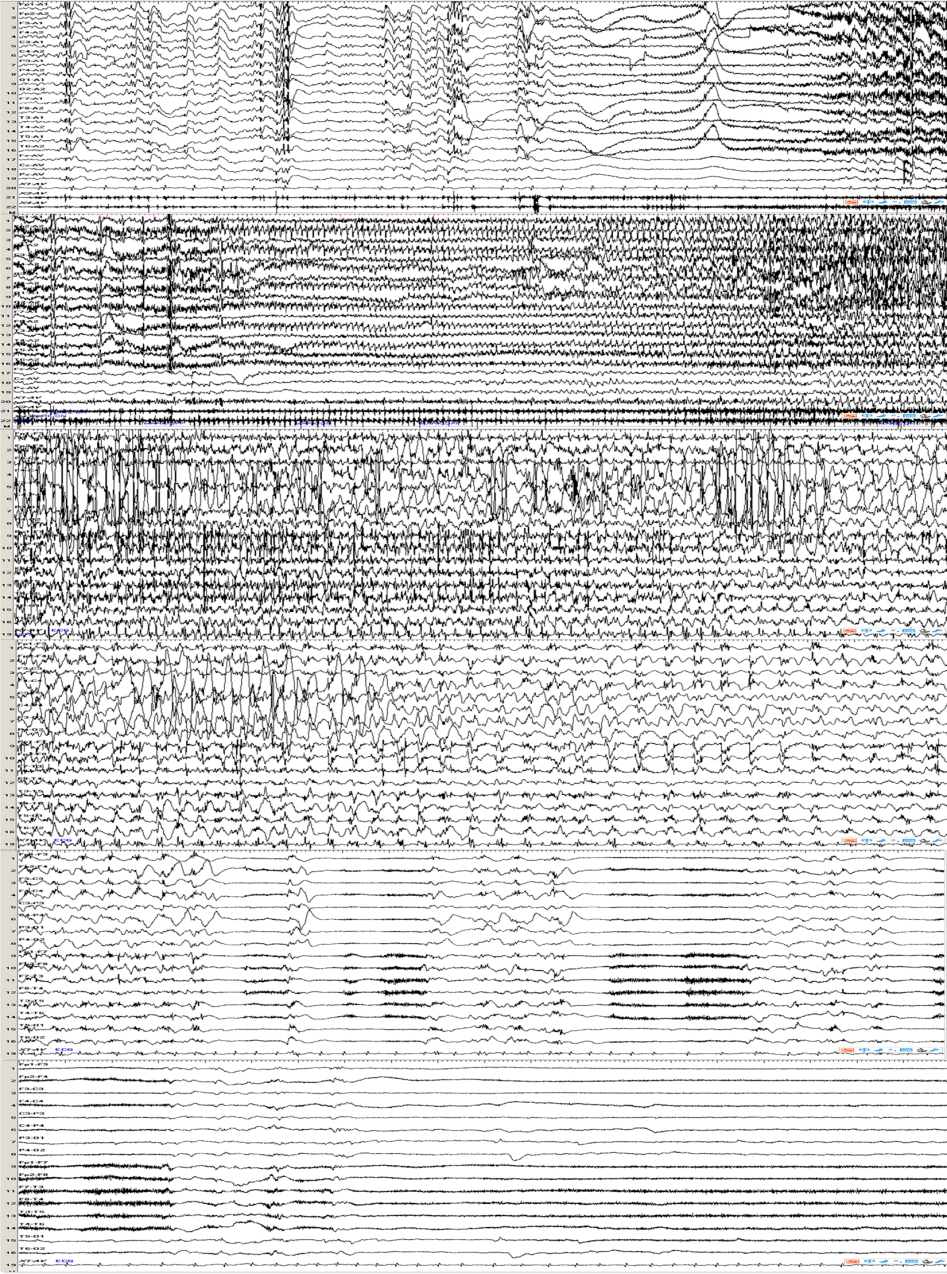
Generalized tonic-clonic seizure preceded by a series of myoclonic seizures, which was evoked by eye closed intermittent photic stimulation at 16 Hz in patient 2. The patient presented as rigidity followed by limbs clonus. The ictal EEG of GTCS showed generalized voltage attenuation lasting for a few seconds, which was followed by generalized spike waves rhythm combined with a large number of muscle artifacts.

**Figure 4:**
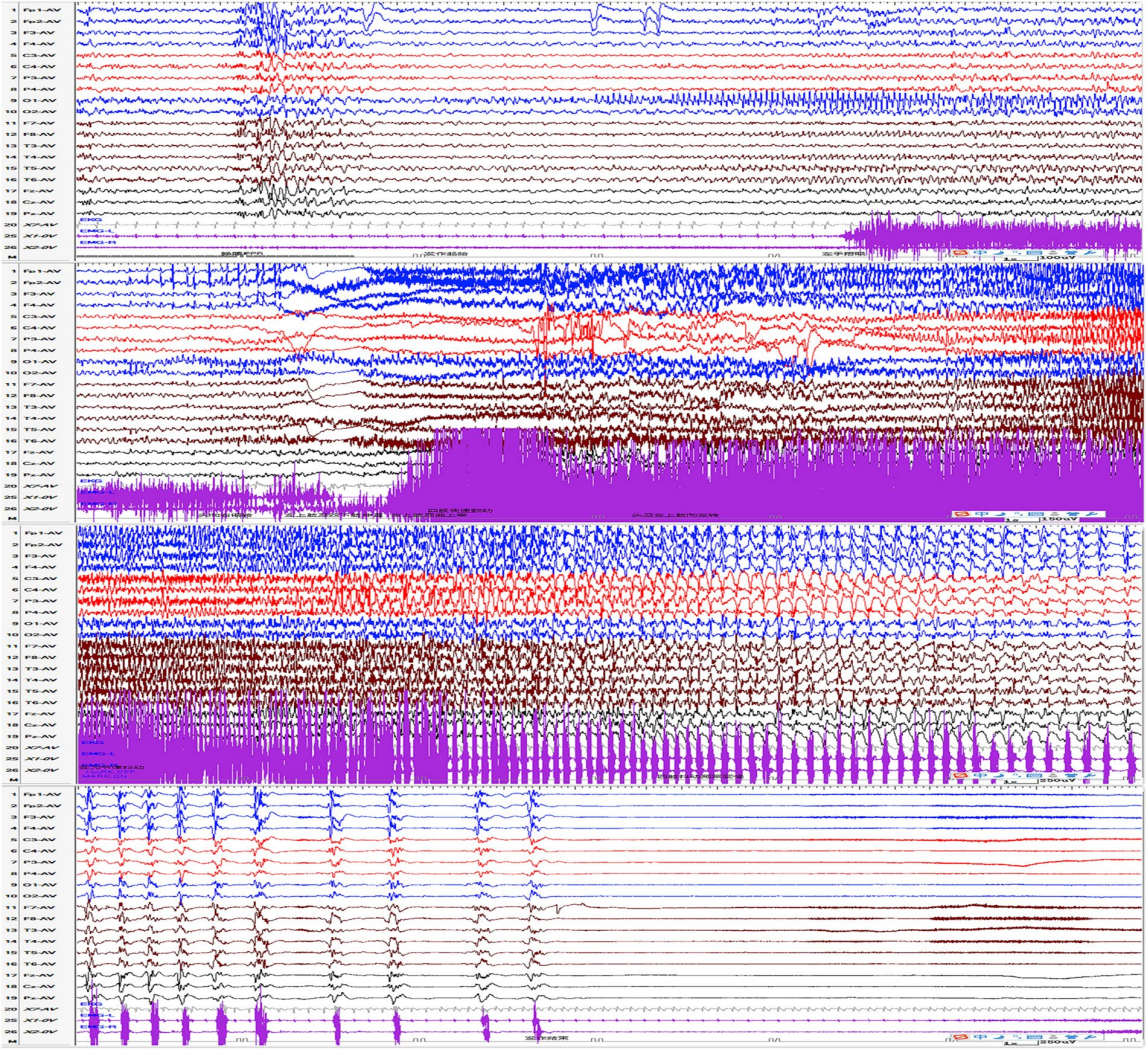
Partial secondarily generalized tonic-clonic seizure evoked by eye opened intermittent photic stimulation at 25 Hz in patient 10. The patient presented as covering eyes with left hand, then right deviation of head, followed by rhythmicity jitter of the right upper limb, stiff of bilateral lower limbs, and then gradually evolved to generalized limbs clonus. The ictal EEG showed rhythmic activities limited to left occipital region and then gradually evolving to generalized.

**Figure 5:**
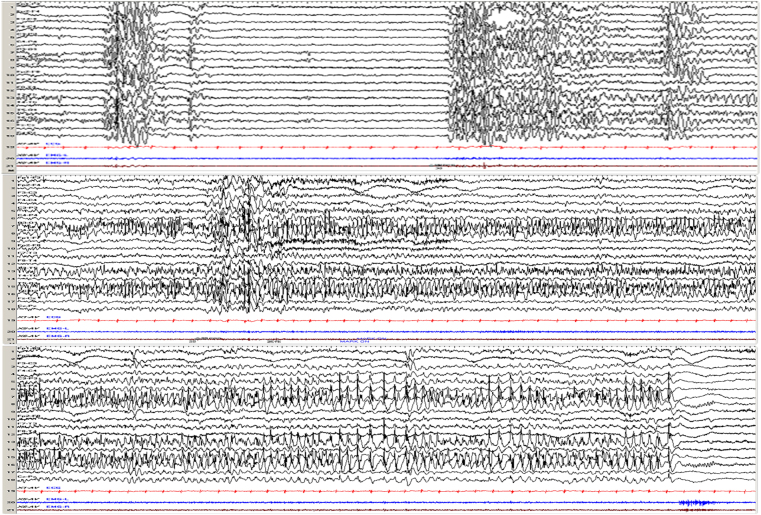
Partial seizure evoked by eye closure intermittent photic stimulation at 30 Hz in patient 14. The patient presented as eyes staring at the right with head turning to right slightly. The ictal EEG showed slow waves in left occipital and posterior temporal areas lasting for about 3 s, followed by spike waves rhythm in left occipital and posterior temporal areas lasting for about 1 min.

**Figure 6:**
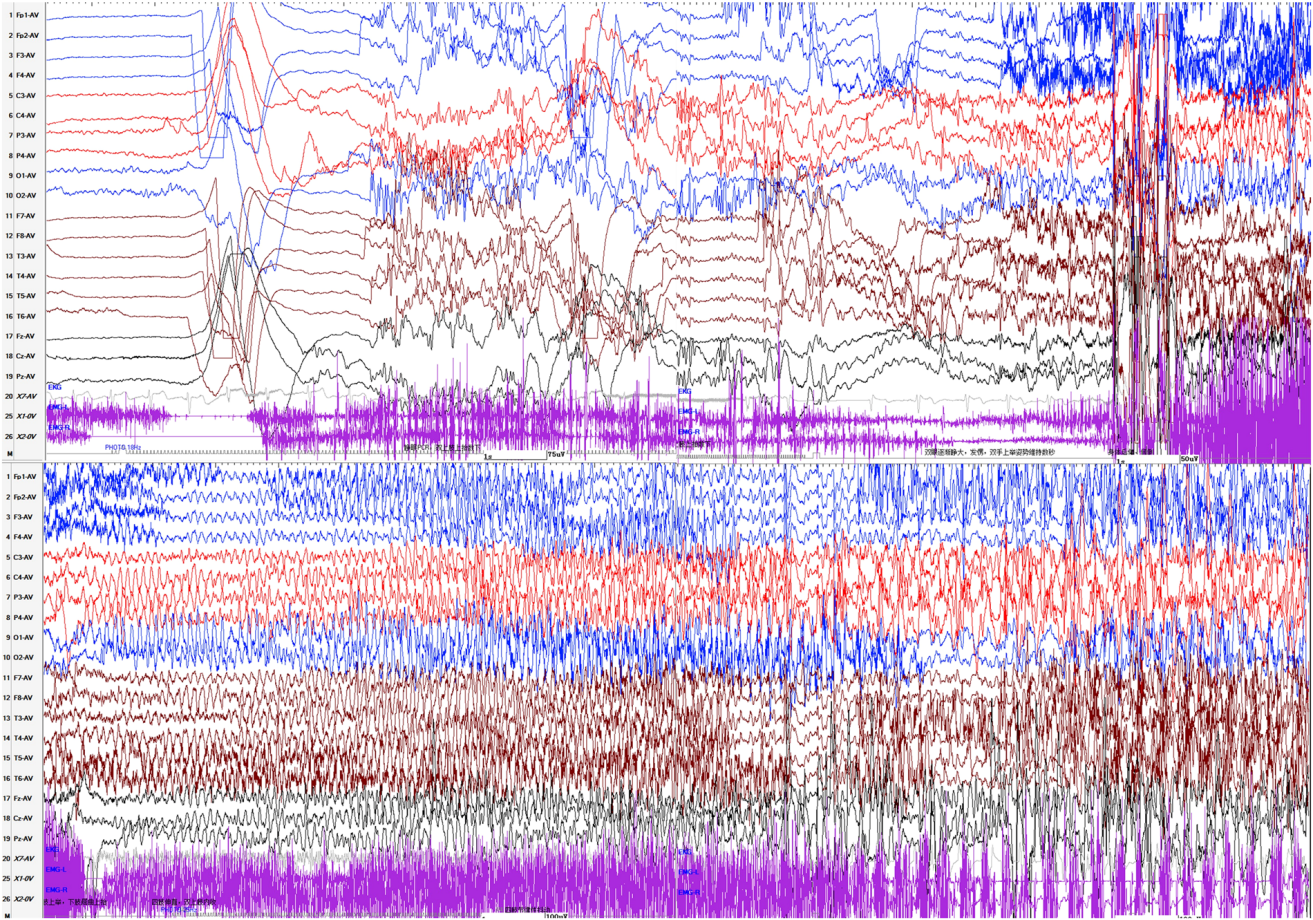
The seizure evoked by eye opened intermittent photic stimulation at 30 Hz in patient 6. The patient presented as staring, followed by generalized limbs stiff and clonus. The ictal EEG showed possible posterior area onset without definite side, and then the discharges gradually spread to the nearby areas and evolved into generalization, which lasted for about 50 s (not fully shown here).

The GTCS induced by IPS in patient 2 presented as rigidity followed by limbs clonus, the ictal EEG showed generalized voltage attenuation lasting for a few seconds, which was followed by generalized spike waves rhythm combined with a large number of muscle artifacts (Fig. 3). The PGTCS induced by IPS presented as consciously eyes and/or head deviation (left or right) followed by generalized limbs convulsions and loss of consciousness, and the corresponding EEG showed rhythmic activities limited to left or right occipital region and then gradually evolving to generalized, with associated gradual increment in amplitude and gradual slowing of frequency from theta to delta range, and then admixed with repetitive spike-wave complexes (Fig. 4). The PS induced by IPS showed eyes and/or head deviation (left or right), staring or nausea clinically, and the ictal EEG showed rhythmic activities of alpha frequency in left or right occipital region with associated gradual increment in amplitude and gradual slowing of frequency, and then admixed with repetitive spike-wave complexes, without generalizing (Fig. 5). The EEG of ES was similar to that of PS, without corresponding clinical presentations. Among the 15 patients with IPS evoked PGTCS or PS, eyes and/or head deviation was observed in 9 patients, staring in 4 patients, nausea in 2 patients, eyes blinking in 2 patients, covering face with hands in 2 patients, and the above presentations could occur in combination. Moreover, 3 patients (patient 6, 7, 12) were described as GTCS/PGTCS (Fig. 6). They all presented as staring, with one patient associated with right arm jerks (patient 7), followed by generalized limbs stiff and clonus. The ictal EEG of patient 7 showed generalized voltage attenuation lasting for 30 seconds without definite focal triggering discharges. And the ictal EEG of patient 6 and 12 showed possible posterior area onset without definite side, and then the discharges gradually spread to the nearby areas and evolved into generalization. It was difficult to determine the occipital region onset or lead-in time in them.

### Diagnosis of epilepsy and epilepsy syndrome

According to the medical history, five patients were considered as idiopathic occipital epilepsy (IOE); sixteen patients were genetic (idiopathic) generalized epilepsy (GGE), including one had JS and another one had MEI. In addition, in patient 22, no afebrile seizure was observed before, she received VEEG examination for febrile seizures with a frequency of once a year.

According to the medical history and VEEG results, the spontaneous “PGTCS” or “GTCS” was revised to be consistent with PCR, and then the electroclinical syndromes were further revised as follows: (1) I(P)OE: spontaneous PS, “PGTCS”, “GTCS” and IPS evoked PS/PGTCS/ES in 12 patients (patient 8–11, 13, 15, 16–21). We used I(P)OE instead of IPOE here, because the visual sensitivity was only found during VEEG monitoring and never be described in the medical histories of all patients. (2) GGE: spontaneous “PGTCS” and IPS evoked GTCS and myoclonic seizures in patient 2. (3) GGE/I(P)OE: spontaneous “GTCS” (combined with eyelid myoclonus in one) and IPS evoked PS/PGTCS/ES combined with myoclonic seizures in 4 patients (patient 1, 3–5); spontaneous myoclonic seizures and IPS evoked PS in patient 14. (4) Pure photosensitive seizure: febrile seizures and IPS evoked PS in patient 22. (5) Epilepsy with undetermined generalized or focal seizure in 3 patients (patient 6, 7, 12): spontaneous “GTCS” (combined with myoclonus in patient 12) and IPS evoked seizures that could not be identified as either GTCS or PGTCS (combined with myoclonus in patient 6).

### Treatment

Nine patients did not receive any treatment of antiepileptic drugs (AEDs), and the other 13 patients were treated by 1 to 5 kinds of AEDs, including valproic acid in 11, lamotrigine in 6, levetiracetam in 3, oxcarbazepine/carbamazepine in 3, clonazepam/nitrazepam in 2, phenobarbital in 2, and topiramate in one. Nineteen patients had normal psychomotor development, three patients (patient 3, 8, 17) had mild cognitive or behavioral deficits, including 2 patients (patient 3, 17) who were diagnosed as attention deficit hyperactivity disorder (ADHD).

## Discussion

The modern environment is a rich resource of seizure triggering visual stimuli. The pathophysiology of photosensitivity is still remained unclear. In the clinical practice, we encountered a problem: some patients with photosensitive seizures, especially those evoked by IPS, were difficult to be diagnosed as the defined epileptic syndromes such as JME, IPOE and so on. In this study, we reported a cohort of 22 patients in whom photosensitive seizures were identified by PCRs during VEEG monitoring. We analyzed clinical features of epilepsy with photosensitive seizures and discussed the probable optimal diagnosis of these patients.

A significant but unexplained association between female sex and photosensitivity had been well-established^13,14^.This was confirmed by the nearly 2:1 preponderance of female in our study. Taylor *et al*.^3,15^ had reported that the inheritance pattern in these patients did not conform to mitochondrial or sex-linked inheritance, and had proved that variations in the photopigment genes on the X chromosome did not account for these sex based differences. Other possible explanations such as hormonal influences or the protective effect of the Y chromosome should be further verified^16^.

Compared to other eye conditions such as eye opened or eye closed, eye closure IPS was easier to evoke PPR^12^. However, in the present study, PPRs or PCRs evoked by eye closure IPS were not more than those evoked by eye opened or eye closed IPS. This was because eye closure IPS was performed at the last for its relatively high risk of inducing seizures, and the procedure would be stopped once seizure evoked by the preceding eye opened or eye closed IPS. The range of flashing frequency inducing PPRs and/or PCRs varied among patients. In most patients here, the range was predominantly 10–20 Hz and was usually overlapped under different eye conditions, which was consistent with the previous report^12^.

Wolf *et al*.^9^ reported that photosensitive epilepsy usually included generalized seizures such as GTCS, absences and myoclonic seizures. PS triggered by visual stimuli were considered uncommon^10^. Whereas, in our cohort, only one patient presented as GTCS induced by IPS, which was not consistent with the previous reports. The reason might be that more focal elements could be found due to the development of monitoring equipment. Fifteen of 22 patients (68.2%) presented as unilateral occipital region onset partial seizures with or without generalizing (PS or PGTCS), as well as 3/22 patients presented as ES initiated from occipital region without clinical presentations. This indicated that occipital cortex might play an important role in the development of photosensitive seizures through initiating the epileptic network. Studies had reported that, occipital seizures, always photic-induced, manifesting as simple visual hallucinations, often with conscious tonic head and eye version, was an integral feature of IPOE^17^. However, simple visual hallucinations were not described in any patients here. There were some probable reasons to explain this phenomenon: lack of visual symptoms actually; the visual symptoms could not be described by some little children; or even, it was hard to be found or was neglected by clinicians and parents during the history-taking.

In the medical records, a total of five seizure types were described, including “GTCS” (13/22), “PGTCS” (3/22), PS (3/22), myoclonic seizures (2/22) and eyelid myoclonus (1/22). According to this, epilepsies in all our patients were divided into IOE and GGE. There should be no problem with the judgment of PS, myoclonic seizures and eyelid myoclonus. For the differentiation of GTCS and PGTCS, due to the retrospective information collection and lack of identification of VEEG, it should be considered more comprehensive. For example, we should consider that whether the “GTCS” was true, or some sensory or motor symptoms of focal seizures were unable to be expressed by the children or were neglected by parents and clinicians. Similarly, we should also consider that whether the “PGTCS” was true, or it was GTCS actually, but had focal or asymmetric presentations at the onset. After combining the seizure types observed in daily life with those captured by VEEG, the diagnosis of electroclinical syndrome was mainly revised as I(P)OE, GGE, and GGE/I(P)OE. The revision was based on the reasons that, in most patients (14/22), the seizure types observed in PCRs were inconsistent with those described in medical records. However, in their parents’ description, there was no difference in the clinical presentations between these two episodes. Therefore, the types of spontaneous seizures were more likely to be same with those captured during IPS essentially.

Classification of the epilepsies into distinct electroclinical syndromes has been one of the most significant achievements of modern epileptology. In our patients, the diagnosis involved from GGEs to mixed GGE/I(P)OE to pure I(P)OE. The co-occurrence of focal and generalized epileptiform discharges, as well as focal and generalized seizure types within one syndrome were not novel. For example, family studies by Taylor *et al*.^18^ had demonstrated phenotypic overlap between IPOE and JME. Then, the same author expanded the finding of overlap between IPOE and GGEs more broadly, including JME, epilepsy with generalized tonic-clonic seizures alone, and childhood absence epilepsy^3^. As both entities of I(P)OE and GGEs were likely to follow polygenic inheritance, the presence of an overlap between them suggested that they might share genetic determinants. Our relatively high rate of patients (31.8%, 7/22) with a family history of febrile seizures or epilepsy provided evidences for this assumption. Perhaps each condition arose from a number of epilepsy genes and various combinations resulted in phenotypic differences, or probably influenced by environmental factors. The pathophysiologic basis of this co-occurrence of focal and generalized seizures within one syndrome remained to be fully explored. It might be explained by the thalamocortical system^18^, which seemed to be involved in the generation of both focal and generalized seizures^17^. In photosensitive patients, visual cortical involvement was reflected by early visual symptoms, promptly followed by phenomena due to discharge spread, especially to motor areas^19,20^. This could explain the finding of conscious eyes and/or head turning and subsequent generalized phenomena seen in our patients. Similarly, early involvement of subcortical structures might be responsible for generalized manifestations^20^. In patients with IPOE and GGE overlap, visual stimuli could trigger abnormal activation of this thalamocortical network, whose variable involvement could generate different (focal and/or generalized) electro-clinical features in the same patient^21^. This mechanism had also been clarified in patients with JS, another idiopathic photosensitive epileptic syndrome, in whom the seizures induced by photic stimulation raised the possibility of the occipital cortex initiating generalized epilepsy network involving the brainstem, thalamocortical and transcortical pathways^22^. Moreover, the IPS-induced focal seizure in one patient supported the role of the visual cortex as a “seizure generator” in JS^23^. Additionally, the recognition of a specific cortical area, in the context of diffuse cortical hyperexcitability, was not uncommon in other idiopathic epilepsies^23^. Therefore, similarly to those suggested in JS by Giráldez *et al*.^23^, epilepsy with photosensitive seizures might be probably better categorized as “systemic epilepsy” rather than as a generalized or focal epilepsy. In another word, the brainstem and thalamocortical network might have reciprocal ways to assist the development of focal and generaliazed seizures, with a modulating switch in the occipital cortex.

In 2010, the International League Against Epilepsy (ILAE) Commission on Classification and Terminology had revised the concepts of generalized and focal seizures as that “Generalized and focal are redefined for seizures as originating at some point within, and rapidly engaging, bilaterally distributed networks (generalized) and originating within networks limited to one hemisphere and either discretely localized or more widely distributed (focal)”^24^. The PCRs in 3 patients here were described as GTCS/PGTCS due to their amphibolous electro-clinical presentations. The clinical symptoms such as staring and unilateral arm jerks, as well as the possibility of occipital region onset in EEG, supported their essence of partial onset. Whereas, the spontaneous “GTCS” or myoclonic seizures during disease courses and the EEG presentations of unclear initial location or sides, made the possibility of generalized seizures could not be ruled out. If they were recognized as GTCS, patient 7 was thought as generalized seizures with some asymmetric presentations at onset, and the other 2 patients were recognized as onset from a point in occipital region and then generalized rapidly. Conversely, if they were recognized as PGTCS, the asymmetric presentations in patient 7 were evidences of focal onset, though the EEG showed generalized voltage attenuation without definite focus; and the other 2 patients were thought as onset at occipital region according to the EEG presentation, though no definite side could be identified and no focal clinical presentations was observed. Previously, in a series of GGE patients, focal interictal epileptiform discharges and semiologic features of focal onset had been observed in 35% patients population^25^. Several authors had also reported that, as seen in secondary GTCS, similar focal features including forced head version, eye version, focal clonic activity, asymmetry in tonic and clonic phases, hemiconvulsion, fencing posture, and unilateral tonic/dystonic posturing could be seen in GTCS of GGE^26–29^. Thus, almost no focal clinical or electrical features recognized generally could identify the diagnosis of focal seizures. If so, the simple dichotomy between focal and generalized seizures might have out of date. The 2017 classification of seizure types by the ILAE divided seizures into focal, generalized and those of unknown onset^30^, which confirmed our findings here. Focal and generalized seizures evoked by visual stimuli might share the epilepsy network involving occipital cortex, brainstem and thalamocortical, in which once seizure initiating from one point, the different diffusion speeds and diffusion ranges lead to the so-called focal or generalized evolution, or unknown onset. It seemed to reinforce the concept that a continuum existed in the spectrum of photosensitive seizures, including focal and generalized seizures as the two endpoints, as well as an intermediate state between them (unknown onset).

Sodium valproate was effective for photosensitive epilepsy^31^, and yielded a good clinical response in the patients with mixed phenotypes of JME/IPOE^18^. Here, 50% (11/22) of the patients used sodium valproate alone or combined with other AEDs. Focal abnormalities in GGEs, as well as generalized discharges in focal epilepsy, might lead to delayed diagnosis and misdiagnosis with inappropriate AEDs choices as a result. The present study proposed the existence of a continuum between focal and generalized seizures in epilepsy with photosensitive seizures, which suggested that, for these patients, it might be more appropriate to use broad spectrum AEDs that were effective for both generalized and focal seizures, such as valproic acid and lamotrigine^32^. The efficacy of AEDs needed to be further evaluated in the future. Psychomotor development was normal in most patients here, excepting mild cognitive or behavioral deficits in 3, which was compatible with the relatively good outcomes in most idiopathic epilepsies.

Our study had several limitations. The retrospective nature limited the acquisition of detailed clinical data, which might induce potential biases; the cohort was too small to draw definitive conclusions; and we were unable to evaluate whether the clinical presentations in our patients were influenced by effect of antiepileptic medications or genetic factors and so on. However, this study took the problems we encountered in clinical practice as a starting point and emphasized on preliminary discussion of pathophysiologic basis of epilepsy with photosensitive seizures, which might attract attentions from more researchers to further study in the future.

## Conclusion

Combination the seizure types observed in daily life and those captured during VEEG, idiopathic epilepsy with photosensitive seizures was classified into I(P)OE, GGE, GGE/I(P)OE, pure photosensitive seizure, and epilepsy with undetermined generalized or focal seizures. The possible explanations were: 1) focal features as an integral component of GGE compatible with that diagnosis; 2) coexistence of both GGE and focal epilepsy in the same patient; 3) the dichotomy might have been out of date regarding to pathophysiological advances in generalized and focal epilepsy. To some extent, it would be better to recognize the idiopathic epilepsy with photosensitive seizures as a continuum between focal and generalized seizures.
